# Supplementary material for: Glucosylsphingosine affects mitochondrial function in a neuronal cell model
Source: Commun Biol. 2025 Aug 21;8:1260. doi: 10.1038/s42003-025-08684-7 (PMC12371010; doi:10.1038/s42003-025-08684-7)
Supplement: Supplementary file 1 — Supplementary Information [file 42003_2025_8684_MOESM1_ESM.pdf]

Supplementary information for the manuscript

Glucosylsphingosine affects mitochondrial function in a neuronal cell model.

Valeria Nikolaenko[1], Reddy Vootukuri[1], Simon Eaton[2], Jenny Hällqvist[1], Tomas Baldwin[1], Kevin Mills[1], Wendy Heywood[1].

[1] Translational Mass Spectrometry Research Group, Genetics & Genomic Medicine Dept., UCL Institute of Child Health, London, WC1N 1EH, UK

[2] Developmental Biology and Cancer Programme, UCL Great Ormond Street Institute of Child Health, London, WC1N 1EH, UK

List of figures

Figure S1. Confirmation of GlcSph entry into cell from culture medium.

Figure S2. The effect of glucosylsphingosine on the activity of alpha enolase.

List of tables

Table S1. Significantly affected canonical pathways by the 20 ng/mL GlcSph exposure in SH-Sy5y cells.

Table S2. Significantly affected canonical pathways by the 200 ng/mL GlcSph exposure in SH-Sye5y cells.

Table S3. Significantly affected canonical pathways in the 20 ng/mL lyso-Gb3 exposed SH-Sy5y cells.

Table S4. Significantly affected canonical pathways in the 200 ng/mL lyso-Gb3 exposed SH-Sy5y cells.

Table S5. Dysregulated proteins identified by the proteomics analysis involved in ATP transport in glucosylsphingosine exposed SH-Sy5y cells.

Table S6. The 54 ubiquitinated proteins exclusive to the glucosylsphingosine dataset.

Supplementary Table S1. Significantly affected canonical pathways by the 20 ng/mL GlcSph exposure in SH-Sy5y cells.

**Canonical Pathways**	**- log (p-value)**	**z-Score**	**Molecules**
Dopamine Receptor Signalling	1.34		COMT, PPP2R1B, PPP2R2A
ILK Signalling	1.34	-0.44	CASP3, MTOR, PPP2R1B, PPP2R2A, RHOG
Phospholipase C Signalling	1.34	1	ARHGEF1, GNA13, GNB2, MPRIP, PLD3, RHOG
Ephrin B Signalling	1.4		CAP1, GNA13, GNB2
Ethanol Degradation II	1.49		ALDH2, ALDH3A2
Mitotic Roles of Polo-Like Kinase	1.55		PPP2R1B, PPP2R2A, RAD21
Systemic Lupus Erythematosus In T-Cell Signalling Pathway	1.57	1.63	CASP3, GNA13, MTOR, PPP2R1B, PPP2R2A, RHOG
Oxidative Phosphorylation	1.62	2	NDUFB11, NDUFB9, NDUFS1, SURF1
Pyrimidine Deoxyribonucleotides De Novo Biosynthesis I	1.66		CMPK1, DTYMK
Role of CHK Proteins in Cell Cycle Checkpoint Control	1.66		ATM, PPP2R1B, PPP2R2A
GDP-L-fucose Biosynthesis I (from GDP-D-mannose)	1.68		GMDS
Glutamine Degradation I	1.68		GLS
L-DOPA Degradation	1.68		COMT
ATM Signalling	1.73	0	ATM, PPP2R1B, PPP2R2A, TRIM28
Ethanol Degradation IV	1.78		ALDH2, ALDH3A2
Valine Degradation I	1.82		DBT, DLD
Putrescine Degradation III	1.87		ALDH2, ALDH3A2
Fatty Acid α-oxidation	1.92		ALDH2, ALDH3A2
Granzyme B Signalling	1.92		CASP3, PRKDC
Oxidative Ethanol Degradation III	1.98		ALDH2, ALDH3A2
BAG2 Signalling Pathway	2.01		ANXA2, CASP3, HSPA8
Colanic Acid Building Blocks Biosynthesis	2.04		GMDS, UGP2
DNA Double-Strand Break Repair by Non-Homologous End Joining	2.04		ATM, PRKDC
Phenylalanine Degradation IV (Mammalian, via Side Chain)	2.04		ALDH2, ALDH3A2
Histamine Degradation	2.1		ALDH2, ALDH3A2
Regulation of eIF4 and p70S6K Signalling	2.24		EIF3K, MTOR, PPP2R1B, PPP2R2A, RPS14, RPS8

Sumoylation Pathway	2.37		RAN, RANBP2, RANGAP1, RCC1, RHOG
Noradrenaline and Adrenaline Degradation	2.43		ALDH2, ALDH3A2, COMT
Huntington's Disease Signalling	2.47		CASP3, DCTN1, GLS, GNB2, HSPA8, MTOR, NAPA, VTI1A
EIF2 Signalling	2.72	2	EIF3K, PTBP1, RPL12, RPL13A, RPL38, RPL6, RPS14, RPS8
Dopamine Degradation	2.76		ALDH2, ALDH3A2, COMT
Acetyl-CoA Biosynthesis I (Pyruvate Dehydrogenase Complex)	2.8		DBT, DLD
Glycine Cleavage Complex	2.8		DLD, GLDC
mTOR Signalling	2.86	-0.42	EIF3K, MTOR, PLD3, PPP2R1B, PPP2R2A, RHOG, RPS14, RPS8
Pyruvate Fermentation to Lactate	2.97		LDHAL6B, LDHB
Tryptophan Degradation X (Mammalian, via Tryptamine)	3.01		ALDH2, ALDH3A2, RDH14
2-ketoglutarate Dehydrogenase Complex	3.19		DLD, OGDH
Branched-chain α-keto acid Dehydrogenase Complex	3.19		DBT, DLD
Phenylethylamine Degradation I	3.19		ALDH2, ALDH3A2
Tight Junction Signalling	3.41		CPSF2, CPSF3, CSTF2, NAPA, PPP2R1B, PPP2R2A, RAB13, VTI1A
Cleavage and Polyadenylation of Pre-mRNA	3.63		CPSF2, CPSF3, CSTF2
Sirtuin Signalling Pathway	3.68	0	GABARAPL2, GLS, H1FX, LDHB, MTOR, NDUFB11, NDUFB9, NDUFS1, PRKDC, TRIM28, VDAC3
Mitochondrial Dysfunction	4.2		ACO2, CASP3, NDUFB11, NDUFB9, NDUFS1, OGDH, PRDX3, SURF1, VDAC3
RAN Signalling	6.17		RAN, RANBP2, RANGAP1, RCC1, XPO1
TCA Cycle II (Eukaryotic)	6.85	2.45	ACO2, DLD, FH, MDH2, OGDH, SUCLA2

Supplementary Table S2. Significantly affected canonical pathways by the 200 ng/mL GlcSph exposure in SH-Sye5y cells.

**Canonical Pathways**	**-log (p-value)**	**z-Score**	**Molecules**
tRNA Charging	6.74	0.71	HARS, HARS2, MARS, QARS, SARS, TARS, VARS, YARS
Protein Ubiquitination Pathway	6.15		CUL1, DNAJA1, HLA-A, HSPA14, HSPA1L, HSPA2, HSPA4, PSMA7, PSMC3, PSMD1, PSMD12, PSMD3, PSMD6, UBB, UBE2N, USP47,
BAG2 Signalling Pathway	4.17	0	ANXA2, HSPA14, HSPA1L, HSPA2, HSPA4, MAPK1
Colanic Acid Building Blocks Biosynthesis	4.16	2	GALE, GMDS, GPI, UGP2
Mitochondrial Dysfunction	3.95		AIFM1, COX6B1, CYB5R3, GSR, HTRA2, NCSTN, NDUFB1, NDUFB9, TXN2, UQCRB, VDAC1
Phagosome Maturation	3.89		ATP6V1A, ATP6V1F, DYNLL1, DYNLT1, HLA-A, PRDX2, RAB5B, VAMP2, VPS18, VTI1A
Huntington's Disease Signalling	3.76		AP2A2, DCTN1, DNM1L, HDAC1, HSPA14, HSPA1L, HSPA2, HSPA4, MAPK1, POLR2E, UBB, VAMP2, VTI1A
Sirtuin Signalling Pathway	3.48	0	ATG3, GTF3C2, H3F3A/H3F3B, LDHA, MAPK1, NDUFB1, NDUFB9, PGK1, PPID, PRKDC, SLC25A5, TIMM23, VDAC1, XRCC6
Glycolysis I	3.2	2	GAPDH, GPI, PGK1, TPI1
Purine Nucleotides De Novo Biosynthesis II	3.15		GART, GMPS, IMPDH2
Gluconeogenesis I	3.13	1	GAPDH, GPI, ME2, PGK1
Acyl-CoA Hydrolysis	3.04		ACOT9, GNPAT, PPT1
Unfolded protein response	2.64		CEBPZ, HSPA14, HSPA1L, HSPA2, HSPA4
Pyruvate Fermentation to Lactate	2.57		LDHA, LDHAL6B
EIF2 Signalling	2.48	2.23	EIF2AK2, EIF3K, EIF4A1, EIF4E, EIF4G2, MAPK1, RPL15, RPL38, RPLP0, RPS17
Pyrimidine Ribonucleotides De Novo Biosynthesis	2.28	2	CAD, CMPK1, DDX3X, NME7
Sucrose Degradation V (Mammalian)	2.13		TKFC, TPI1
NER Pathway	2.13	-0.44	CHAF1B, COPS3, H3F3A/H3F3B, POLR2E, RPA1, UBE2N
UDP-N-acetyl-D-galactosamine Biosynthesis II	2.03		GALE, GPI
Glucocorticoid Receptor Signalling	1.96		BRD7, HSPA14, HSPA1L, HSPA2, HSPA4, KRT1, KRT10, KRT2, KRT9, MAPK1, POLR2E, SMARCD1
Regulation of eIF4 and p70S6K Signalling	1.81		EIF3K, EIF4A1, EIF4E, EIF4G2, MAPK1, PTPA, RPS17
Clathrin-mediated Endocytosis Signalling	1.79		ACTR2, ALB, AP2A2, AP2M1, CSNK2A1, DNM1L, RAB5B, UBB

CTLA4 Signalling in Cytotoxic T Lymphocytes	1.79		AP2A2, AP2M1, HLA-A, PTPA, PTPN11
Mevalonate Pathway I	1.78		ACAT1, IDI1
UDP-N-acetyl-D-galactosamine Biosynthesis I	1.77		GALE
DNA Methylation and Transcriptional Repression Signalling	1.72		CHD4, H3F3A/H3F3B, HDAC1
Mitotic Roles of Polo-Like Kinase	1.67		ANAPC7, CDC27, PTPA, RAD21
mTOR Signalling	1.66	0	EIF3K, EIF4A1, EIF4E, EIF4G2, MAPK1, PLD3, PTPA, RPS17
Salvage Pathways of Pyrimidine Ribonucleotides	1.66	2.23	CMPK1, EIF2AK2, MAPK1, NME7, PAK1
DNA Double-Strand Break Repair by Non-Homologous End Joining	1.65		PRKDC, XRCC6
Tight Junction Signalling	1.64		AFDN, CPSF3, CTNNA1, PTPA, SYMPK, VAMP2, VTI1A
Remodelling of Epithelial Adherens Junctions	1.6		ACTR2, CTNNA1, DNM1L, RAB5B
Hereditary Breast Cancer Signalling	1.55		BRD7, HDAC1, POLR2E, RPA1, SMARCD1, UBB
Oestrogen Receptor Signalling	1.55		H3F3A/H3F3B, MAPK1, PELP1, POLR2E, PRKDC, TRRAP
Superpathway of Geranylgeranyldiphosphate Biosynthesis I (via Mevalonate)	1.54		ACAT1, IDI1
Pyrimidine Ribonucleotides Interconversion	1.53		CMPK1, DDX3X, NME7
β-alanine Degradation I	1.48		ABAT
Uridine-5'-phosphate Biosynthesis	1.48		CAD
GDP-L-fucose Biosynthesis I (from GDP-D-mannose)	1.48		GMDS
Formaldehyde Oxidation II (Glutathione-dependent)	1.48		ADH5
Sertoli Cell-Sertoli Cell Junction Signalling	1.47		AFDN, CLINT1, CTNNA1, MAPK1, PLS1, SPTBN1, SYMPK
Caveolar-mediated Endocytosis Signalling	1.46		ALB, COPG2, HLA-A, RAB5B
Synaptogenesis Signalling Pathway	1.45	2.33	ACTR2, AFDN, AP2A2, AP2M1, MAPK1, NAP1L1, PAK1, RAB5B, VAMP2, VTI1A
Stearate Biosynthesis I (Animals)	1.43		ACOT9, GNPAT, PPT1
Toll-like Receptor Signalling	1.43		EIF2AK2, MAPK1, TOLLIP, UBB
The Visual Cycle	1.4		RBP1, RDH11
3-phosphoinositide Degradation	1.4	1.63	ILKAP, MTMR1, PPIP5K1, PTPA, PTPN11, UBLCP1
Renal Cell Carcinoma Signalling	1.37		MAPK1, PAK1, PTPN11, UBB

RAR Activation	1.37		BRD7, CSNK2A1, MAPK1, RBP1, RDH11, SMAD9, SMARCD1
Role of BRCA1 in DNA Damage Response	1.34		BRD7, E2F4, RPA1, SMARCD1
Cyclins and Cell Cycle Regulation	1.34		CUL1, E2F4, HDAC1, PTPA
Aldosterone Signalling in Epithelial Cells	1.34		DNAJA1, HSPA14, HSPA1L, HSPA2, HSPA4, MAPK1
Role of Tissue Factor in Cancer	1.34		EIF4E, GNA13, MAPK1, PAK1, PTPN11
HOTAIR Regulatory Pathway	1.32	0.44	ATG3, H3F3A/H3F3B, HDAC1, KDM1A, SUZ12, TCF4
NADH Repair	1.31		GAPDH
Methylglyoxal Degradation I	1.31		GLO1
5-aminoimidazole Ribonucleotide Biosynthesis I	1.31		GART
Coenzyme A Biosynthesis	1.31		COASY
4-aminobutyrate Degradation I	1.31		ABAT

Supplementary Table S3. Significantly affected canonical pathways in the 20 ng/mL lyso-Gb3 exposed SH-Sy5y cells.

**Canonical Pathways**	**-log (p-value)**	**z-Score**	**Molecules**
ErbB2-ErbB3 Signalling	1.31		GRB2, MAP2K1
Regulation of Cellular Mechanics by Calpain Protease	1.31		EZR, GRB2
UDP-N-acetyl-D-galactosamine Biosynthesis II	1.31		GPI
Colorectal Cancer Metastasis Signalling	1.31	1	GRB2, MAP2K1, PRKAR2B, TCF4
Mitotic Roles of Polo-Like Kinase	1.32		ANAPC7, PPP2R2A
Thrombopoietin Signalling	1.32		GRB2, MAP2K1
Relaxin Signalling	1.32		GNAI2, MAP2K1, PRKAR2B
Opioid Signalling Pathway	1.32	1	AP2B1, GNAI2, MAP2K1, PRKAR2B
Role of PI3K/AKT Signalling in the Pathogenesis of Influenza	1.34		GNAI2, MAP2K1
Signalling by Rho Family GTPases	1.35		EZR, GNAI2, MAP2K1, VIM
Corticotropin Releasing Hormone Signalling	1.36		GNAI2, MAP2K1, PRKAR2B
Endometrial Cancer Signalling	1.36		GRB2, MAP2K1
Cardiac β-adrenergic Signalling	1.37		PPP1R10, PPP2R2A, PRKAR2B
Ovarian Cancer Signalling	1.38		MAP2K1, PRKAR2B, TCF4
Role of CHK Proteins in Cell Cycle Checkpoint Control	1.4		ATM, PPP2R2A
PI3K Signalling in B Lymphocytes	1.41		MAP2K1, PDIA3, SYK
CNTF Signalling	1.41		GRB2, MAP2K1
Aspartate Degradation II	1.42		GOT2
PI3K/AKT Signalling	1.44		GRB2, MAP2K1, PPP2R2A
Endocannabinoid Neuronal Synapse Pathway	1.47		GNAI2, PDIA3, PRKAR2B
PTEN Signalling	1.48		CSNK2A1, GRB2, MAP2K1
Gαi Signalling	1.49		GNAI2, GRB2, PRKAR2B
IL-6 Signalling	1.49		CSNK2A1, GRB2, MAP2K1
Amyloid Processing	1.5		CSNK2A1, PRKAR2B
Thyroid Cancer Signalling	1.5		MAP2K1, TCF4
Actin Cytoskeleton Signalling	1.51	0	EZR, GRB2, MAP2K1, PFN1
G Beta Gamma Signalling	1.51		GNAI2, GRB2, PRKAR2B

Sperm Motility	1.52		MAP2K1, PDIA3, PRKAR2B, SYK
Fc Epsilon RI Signalling	1.56		GRB2, MAP2K1, SYK
Natural Killer Cell Signalling	1.56		GRB2, MAP2K1, SYK
Renin-Angiotensin Signalling	1.56		GRB2, MAP2K1, PRKAR2B
Creatine-phosphate Biosynthesis	1.56		CKMT1A/CKMT1B
Pyruvate Fermentation to Lactate	1.56		LDHAL6B
Thrombin Signalling	1.57	1	GNAI2, GRB2, MAP2K1, PDIA3
CD28 Signalling in T Helper Cells	1.58		GRB2, MAP2K1, SYK
Endocannabinoid Developing Neuron Pathway	1.58		GNAI2, MAP2K1, PRKAR2B
GPCR-Mediated Nutrient Sensing in Enteroendocrine Cells	1.62		GNAI2, PDIA3, PRKAR2B
Oncostatin M Signalling	1.62		GRB2, MAP2K1
BAG2 Signalling Pathway	1.64		ANXA2, HSPA9
Adrenomedullin signalling pathway	1.65	1	GRB2, MAP2K1, PDIA3, PRKAR2B
Glutathione Redox Reactions II	1.66		PDIA3
L-cysteine Degradation I	1.66		GOT2
Pentose Phosphate Pathway (Oxidative Branch)	1.66		G6PD
Sirtuin Signalling Pathway	1.68		G6PD, GOT2, POLR1C, TRIM28, TUBA1B
Molecular Mechanisms of Cancer	1.69		ATM, GNAI2, GRB2, MAP2K1, PRKAR2B, TCF4
Telomerase Signalling	1.69		GRB2, MAP2K1, PPP2R2A
Mouse Embryonic Stem Cell Pluripotency	1.71		GRB2, MAP2K1, TCF4
Synaptic Long Term Depression	1.73	1	GNAI2, MAP2K1, PDIA3, PPP2R2A
Acute Phase Response Signalling	1.76		C4A/C4B, GRB2, MAP2K1, TCF4
Role of NFAT in Regulation of the Immune Response	1.77	0	GNAI2, GRB2, MAP2K1, SYK
Aspartate Biosynthesis	1.78		GOT2
Glutamate Degradation II	1.78		GOT2
ATM Signalling	1.78		ATM, PPP2R2A, TRIM28
Axonal Guidance Signalling	1.81		GNAI2, GRB2, MAP2K1, PDIA3, PFN1, PRKAR2B, TUBA1B
Melanocyte Development and Pigmentation Signalling	1.81		GRB2, MAP2K1, PRKAR2B
α-Adrenergic Signalling	1.82		GNAI2, MAP2K1, PRKAR2B

GNRH Signalling	1.82	1	GNAI2, GRB2, MAP2K1, PRKAR2B
Acute Myeloid Leukaemia Signalling	1.88		GRB2, MAP2K1, TCF4
PDGF Signalling	1.91		CSNK2A1, GRB2, MAP2K1
BMP signalling pathway	1.95		GRB2, MAP2K1, PRKAR2B
Fatty Acid Biosynthesis Initiation II	1.95		FASN
GDP-L-fucose Biosynthesis I (from GDP-D-mannose)	1.95		GMDS
Palmitate Biosynthesis I (Animals)	1.95		FASN
Aldosterone Signalling in Epithelial Cells	1.97		HSPA9, MAP2K1, PDIA3, TRAP1
Cardiac Hypertrophy Signalling	1.99	0.45	GNAI2, GRB2, MAP2K1, PDIA3, PRKAR2B
Dopamine Receptor Signalling	2.06		PPP1R10, PPP2R2A, PRKAR2B
Gluconeogenesis I	2.07		ENO2, GPI
Glycolysis I	2.11		ENO2, GPI
Lipid Antigen Presentation by CD1	2.11		AP2B1, PDIA3
GPCR-Mediated Integration of Enteroendocrine Signalling Exemplified by an L Cell	2.12		GNAI2, PDIA3, PRKAR2B
Insulin Receptor Signalling	2.16	0	GRB2, MAP2K1, PPP1R10, PRKAR2B
Role of NFAT in Cardiac Hypertrophy	2.18	0.45	GNAI2, GRB2, MAP2K1, PDIA3, PRKAR2B
CREB Signalling in Neurons	2.24	1.34	GNAI2, GRB2, MAP2K1, PDIA3, PRKAR2B
Protein Kinase A Signalling	2.25	0	ANAPC7, GNAI2, MAP2K1, PDIA3, PPP1R10, PRKAR2B, TCF4
P2Y Purigenic Receptor Signalling Pathway	2.27	1	GNAI2, MAP2K1, PDIA3, PRKAR2B
Synaptic Long Term Potentiation	2.27	1	MAP2K1, PDIA3, PPP1R10, PRKAR2B
ERK/MAPK Signalling	2.36	-0.45	GRB2, MAP2K1, PPP1R10, PPP2R2A, PRKAR2B
EGF Signalling	2.45		CSNK2A1, GRB2, MAP2K1
CDK5 Signalling	2.52	1	MAP2K1, PPP1R10, PPP2R2A, PRKAR2B
IGF-1 Signalling	2.56	1	CSNK2A1, GRB2, MAP2K1, PRKAR2B
PFKFB4 Signalling Pathway	2.67		GPI, MAP2K1, PRKAR2B
Dopamine-DARPP32 Feedback in cAMP Signalling	2.71	0.45	GNAI2, PDIA3, PPP1R10, PPP2R2A, PRKAR2B
CTLA4 Signalling in Cytotoxic T Lymphocytes	2.83		AP2B1, GRB2, PPP2R2A, SYK
Systemic Lupus Erythematosus In T Cell Signalling Pathway	2.88	-0.82	EZR, GNAI2, GRB2, MAP2K1, PPP2R2A, SYK

Endocannabinoid Cancer Inhibition Pathway	2.92	-0.44	GNAI2, MAP2K1, PRKAR2B, TCF4, VIM
Gap Junction Signalling	3.12		GNAI2, GRB2, MAP2K1, PDIA3, PRKAR2B, TUBA1B
Leptin Signalling in Obesity	3.13		GRB2, MAP2K1, PDIA3, PRKAR2B
14-3-3-mediated Signalling	3.16	1	GRB2, MAP2K1, PDIA3, TUBA1B, VIM
Melatonin Signalling	3.2	2	GNAI2, MAP2K1, PDIA3, PRKAR2B
PPARα/RXRα Activation	3.31	1.34	FASN, GOT2, GRB2, MAP2K1, PDIA3, PRKAR2B
GDP-mannose Biosynthesis	3.34		GMPPB, GPI
IL-2 Signalling	3.43		CSNK2A1, GRB2, MAP2K1, SYK
EIF2 Signalling	3.72	0.82	EIF5, GRB2, MAP2K1, RPL12, RPL27, RPL36, WARS
Breast Cancer Regulation by Stathmin1	3.9		GNAI2, GRB2, MAP2K1, PPP1R10, PPP2R2A, PRKAR2B, TUBA1B
p70S6K Signalling	4.09	0	GNAI2, GRB2, MAP2K1, PDIA3, PPP2R2A, SYK
Colanic Acid Building Blocks Biosynthesis	4.23		GMDS, GMPPB, GPI
tRNA Charging	7.21	1.63	EPRS, FARSB, HARS, MARS, NARS, WARS

Supplementary Table S4. Significantly affected canonical pathways in the 200 ng/mL lyso-Gb3 exposed SH-Sy5y cells.

Ingenuity Canonical Pathways	-log (p-value)	z-Score	Molecules
Protein Ubiquitination Pathway	5.12		CUL1, DNAJA1, HLA-A, HSP90AA1, PSMA6, PSMA7, PSMB1, PSMC3, PSMD1, PSMD12, PSMD3, UBE2N, UBE2V1, USP47
Cleavage and Polyadenylation of Pre-mRNA	4.96		CPSF2, CSTF3, NUDT21, PAPOLA
Regulation of eIF4 and p70S6K Signalling	4.6		EIF2S1, EIF3K, EIF4E, PPP2CB, PPP2R5E, RPS13, RPS17, RPS8, RPS9, RPSA
Sirtuin Signalling Pathway	4.21	0.63	GABARAPL2, GLS, H1FX, LDHB, NDUFB1, NDUFS5, NDUFS8, POLR1B, PRKDC, TOMM70, TRIM28, VDAC3, XRCC6
EIF2 Signalling	4.09		EIF2S1, EIF3K, EIF4E, RPL15, RPL27, RPL27A, RPS13, RPS17, RPS8, RPS9, RPSA
TCA Cycle II (Eukaryotic)	3.68	2	ACO2, FH, IDH3G, SUCLG1
mTOR Signalling	3.6	-1	EIF3K, EIF4E, PPP2CB, PPP2R5E, RHOG, RPS13, RPS17 , RPS8, RPS9, RPSA
Cyclins and Cell Cycle Regulation	3.3	-1	ATM, CUL1, E2F4, HDAC2, PPP2CB, PPP2R5E
DNA Double-Strand Break Repair by Non-Homologous End Joining	3.2		ATM, PRKDC, XRCC6
Role of CHK Proteins in Cell Cycle Checkpoint Control	3.14	1	ATM, E2F4, PPP2CB, PPP2R5E, RFC2
RAN Signalling	2.94		RCC1, TNPO1, XPO1
Pyruvate Fermentation to Lactate	2.82		LDHAL6B, LDHB
Breast Cancer Regulation by Stathmin1	2.44		ARHGEF1, E2F4, GNAS, GNB1, PPP2CB, PPP2R5E, TSG10 1, TUBB6
Mitochondrial Dysfunction	2.32		ACO2, FIS1, NDUFB1, NDUFS5, NDUFS8, PRDX3, VDAC3
CTLA4 Signalling in Cytotoxic T Lymphocytes	2.3		AP2M1, HLA-A, PPP2CB, PPP2R5E, SYK
Purine Nucleotides De Novo Biosynthesis II	2.1		GART, GMPS
Mitotic Roles of Polo-Like Kinase	2.1		HSP90AA1, KIF23, PPP2CB, PPP2R5E
Cardiac β-adrenergic Signalling	2.09		AKAP13, AKAP8, GNAS, GNB1, PPP2CB, PPP2R5E
Phagosome Maturation	2.08		ATP6V1A, DYNC1H1, DYNC1I2, HLA-A, TSG101, TUBB6
Sumoylation Pathway	2.06		HDAC2, KDM1A, RCC1, RFC2, RHOG
ILK Signalling	2.05	0	ACTN1, MYH9, PPP2CB, PPP2R5E, RHOG, RSU1, VIM
Cell Cycle: G1/S Checkpoint Regulation	2.03		ATM, CUL1, E2F4, HDAC2
Mevalonate Pathway I	2.02		IDI1, MVK
Cell Cycle Regulation by BTG Family Proteins	1.96		E2F4, PPP2CB, PPP2R5E
Hypoxia Signalling in the Cardiovascular System	1.9		ATM, HSP90AA1, UBE2N, UBE2V1
Colanic Acid Building Blocks Biosynthesis	1.89		GMDS, UGP2

Vitamin-C Transport	1.83		GSTO1, TXNRD1
Superpathway of Geranylgeranyldiphosphate Biosynthesis I (via Mevalonate)	1.78		IDI1, MVK
Tight Junction Signalling	1.73		CPSF2, CSTF3, MYH9, NUDT21, PPP2CB, PPP2R5E
Granzyme A Signalling	1.73		H1FX, HMGB2
Tryptophan Degradation X (Mammalian, via Tryptamine)	1.63		ALDH2, DDC
Cell Cycle: G2/M DNA Damage Checkpoint Regulation	1.63		ATM, CUL1, PRKDC
Spermidine Biosynthesis I	1.6		SRM
GDP-L-fucose Biosynthesis I (from GDP-D-mannose)	1.6		GMDS
Glutamine Degradation I	1.6		GLS
ATM Signalling	1.49	0	ATM, PPP2CB, PPP2R5E, TRIM28
Phospholipase C Signalling	1.45	0.82	ARHGEF1, GNAS, GNB1, HDAC2, MPRIP, RHOG, SYK
Glycolysis I	1.44		ENO1, PKM
Diphthamide Biosynthesis	1.43		EEF2
5-aminoimidazole Ribonucleotide Biosynthesis I	1.43		GART
Ascorbate Recycling (Cytosolic)	1.43		GSTO1
Telomerase Signalling	1.38		HDAC2, HSP90AA1, PPP2CB, PPP2R5E
Gαs Signalling	1.36	0	ADD1, ADD2, GNAS, GNB1
Superpathway of Cholesterol Biosynthesis	1.35		IDI1, MVK
Arsenate Detoxification I (Glutaredoxin)	1.31		GSTO1
Catecholamine Biosynthesis	1.31		DDC
Phenylethylamine Degradation I	1.31		ALDH2
Trans, trans-farnesyl Diphosphate Biosynthesis	1.31		IDI1
Remodelling of Epithelial Adherens Junctions	1.3		ACTN1, DNM1L, TUBB6

Supplementary Table S5. Dysregulated proteins identified by the proteomics analysis involved in ATP transport in glucosylsphingosine exposed SH-Sy5y cells.

Gene	Protein	p-value	Fold change	p-value	Fold change
		20 ng/mL		200 ng/mL	
AK3	adenylate kinase 3	0.121	2.7	0.009	3.6
CKMT1B	creatine kinase, mitochondrial 1B	0.055	1.9	0.003	2.4
GSR	glutathione-disulfide reductase	0.239	-2.2	0.029	-2.9
SLC25A3	solute carrier family 25 member 3	0.027	2.1	0.63	1.3
SLC25A5	solute carrier family 25 member 5	0.233	4.9	0.034	3.2
SLC25A6	solute carrier family 25 member 6	0.348	-2.8	0.078	-3.2
VDAC1	voltage dependent anion channel 1	0.271	4.6	0.015	2.7
VDAC3	voltage dependent anion channel 3	0.026	4.1	0.155	2.7

Supplementary Table S6. The 54 ubiquitinated proteins exclusive to the glucosylsphingosine dataset.

Gene	Protein	Amount (fmol)
TUBA1C	Tubulin alpha-1C chain	7.761
RPL21	60S ribosomal protein L21	0.993
SLC45A4	Solute carrier family 45 member 4	0.782
TUBB1	Tubulin beta-1 chain	0.767
TUBB4A	Tubulin beta-4A chain	0.645
HIST2H2BC	Putative histone H2B type 2-C	0.519
GNB2	Guanine nucleotide-binding protein G(I)/G(S)/G(T) subunit beta-2	0.419
RPS11	40S ribosomal protein S11	0.409
TPT1	Translationally-controlled tumor protein	0.368
PSME2	Proteasome activator complex subunit 2	0.358
RPL34	60S ribosomal protein L34	0.339
GNB1	Guanine nucleotide-binding protein G(I)/G(S)/G(T) subunit beta-1	0.318
DDX39B	Spliceosome RNA helicase DDX39B	0.313
CLIC1	Chloride intracellular channel protein 1	0.310
RPL35	60S ribosomal protein L35	0.306
KRT17	Keratin_ type I cytoskeletal 17	0.297
KRT5	Keratin_ type II cytoskeletal 5	0.294
HBD	Hemoglobin subunit delta	0.281
HBA1	Hemoglobin subunit alpha	0.278
CACYBP	Calcyclin-binding protein	0.247
EIF4A2	Eukaryotic initiation factor 4A-II	0.240
NUTF2	Nuclear transport factor 2	0.239
TAGLN2	Transgelin-2	0.237
PPP2CB	Serine/threonine-protein phosphatase 2A catalytic subunit beta	0.189
HIST1H2AH	Histone H2A type 1-H	0.180
SHMT2	Serine hydroxymethyltransferase_ mitochondrial	0.177
FASN	Fatty acid synthase	0.166
KRT6C	Keratin_ type II cytoskeletal 6C	0.164
HSPA6	Heat shock 70 kDa protein 6	0.163
UBE2L3	Ubiquitin-conjugating enzyme E2 L3	0.152
KRT6B	Keratin_ type II cytoskeletal 6B	0.151
GNAS	Guanine nucleotide-binding protein G(s) subunit alpha isoforms short	0.151
HADHA	Trifunctional enzyme subunit alpha_ mitochondrial	0.144
ECH1	Delta(3_5)-Delta(2_4)-dienoyl-CoA isomerase_ mitochondrial	0.122
OLA1	Obg-like ATPase 1	0.115
APEX1	DNA-(apurinic or apyrimidinic site) lyase	0.113
PNMA2	Paraneoplastic antigen Ma2	0.110
RPL29	60S ribosomal protein L29	0.106
ESD	S-formylglutathione hydrolase	0.098
WDR61	WD repeat-containing protein 61	0.091
PPP1CC	Serine/threonine-protein phosphatase PP1-gamma catalytic subunit	0.087
WDR1	WD repeat-containing protein 1	0.083
APRT	Adenine phosphoribosyltransferase	0.073
PSAT1	Phosphoserine aminotransferase	0.072
KRT26	Keratin_ type I cytoskeletal 26	0.065
RBM39	RNA-binding protein 39	0.065
HSPA2	Heat shock-related 70 kDa protein 2	0.064
ACAA2	3-ketoacyl-CoA thiolase_ mitochondrial	0.056
PGM1	Phosphoglucomutase-1	0.039
MSH2	DNA mismatch repair protein Msh2	0.033
NEFL	Neurofilament light polypeptide	0.033
HSP90AB4P	Putative heat shock protein HSP 90-beta 4	0.021
HSPA1L	Heat shock 70 kDa protein 1-like	0.016
Total		19.816

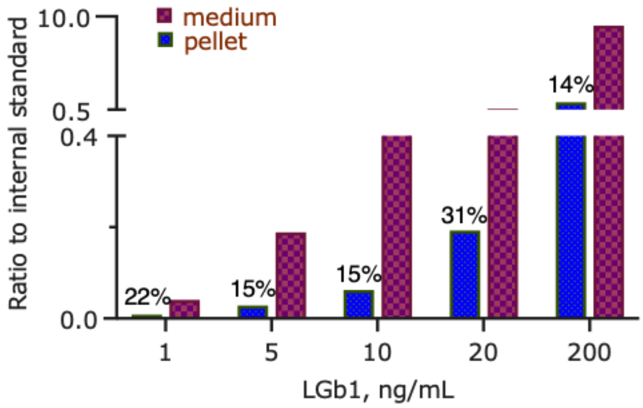

Supplementary Figure S1. Confirmation of GlcSph entry into cells from culture medium supplemented with GlcSph demonstrates proportional increase in cellular uptake of GlcSph with its increasing concentrations in culture medium. Lysate levels are expressed as % of GlcSph levels in culture medium. LGb1=GlcSph. Source data are provided as a Supplementary Data file.

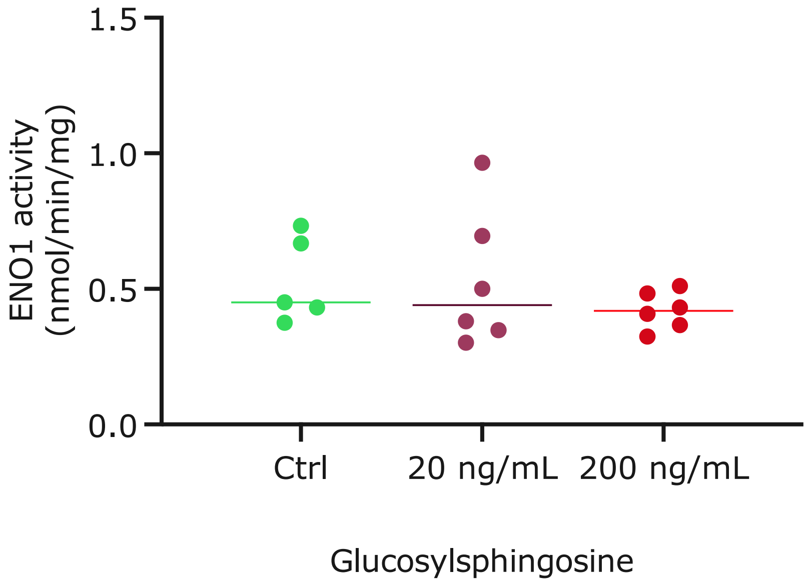

Supplementary Figure S2. The activity of alpha enolase in SH-Sy5y cells treated with 20 ng/mL and 200 ng/mL of GlcSph and vehicle for 72 hours. Data were analysed by the Kruskal-Wallis test followed by the Dunn's multiple comparisons post-hoc test. Data are mean ± SD, (n=6). Source data are provided as a Supplementary Data file.
