# Supplementary material for: Glucosylsphingosine affects mitochondrial function in a neuronal cell model
Source: Commun Biol. 2025 Aug 21;8:1260. doi: 10.1038/s42003-025-08684-7 (PMC12371010; doi:10.1038/s42003-025-08684-7)
Supplement: Supplementary file 4 — Reporting summary [file 42003_2025_8684_MOESM4_ESM.pdf]

Corresponding author(s): Wendy Heywood

Last updated by author(s): Jul 20, 2025

Reporting Summary

Nature Portfolio wishes to improve the reproducibility of the work that we publish. This form provides structure for consistency and transparency in reporting. For further information on Nature Portfolio policies, see our Editorial Policies and the Editorial Policy Checklist.

Statistics

For all statistical analyses, confirm that the following items are present in the figure legend, table legend, main text, or Methods section.

n/a	Confirmed	
☐	☒	The exact sample size (*n*) for each experimental group/condition, given as a discrete number and unit of measurement
☐	☒	A statement on whether measurements were taken from distinct samples or whether the same sample was measured repeatedly
☐	☒	The statistical test(s) used AND whether they are one- or two-sided<br>*Only common tests should be described solely by name; describe more complex techniques in the Methods section.*
☒	☐	A description of all covariates tested
☐	☒	A description of any assumptions or corrections, such as tests of normality and adjustment for multiple comparisons
☐	☒	A full description of the statistical parameters including central tendency (e.g. means) or other basic estimates (e.g. regression coefficient) AND variation (e.g. standard deviation) or associated estimates of uncertainty (e.g. confidence intervals)
☐	☒	For null hypothesis testing, the test statistic (e.g. *F*, *t*, *r*) with confidence intervals, effect sizes, degrees of freedom and *P* value noted<br>*Give P values as exact values whenever suitable.*
☒	☐	For Bayesian analysis, information on the choice of priors and Markov chain Monte Carlo settings
☒	☐	For hierarchical and complex designs, identification of the appropriate level for tests and full reporting of outcomes
☒	☐	Estimates of effect sizes (e.g. Cohen's *d*, Pearson's *r*), indicating how they were calculated

Our web collection on statistics for biologists contains articles on many of the points above.

Software and code

Policy information about availability of computer code

Data collection	Discovery mass spectrometric data were collected by the vendor software MassLynx (v. 4.1). Peak picking, integrations and protein identifications were performed in the vendor software Progenesis QI for proteomics (v. 31.46.2004), for IP-LCMS data to identify ubiquitinated proteins and lipid binding proteins protein identifications were performed in the vendor software PLGS (v.3.03). Oxygen consumption and extracellular acidification rates for ATP production and glycolytic rates in live cells were collected using vendor instrument Seahorse XFp (Agilent) and software Wave (v. 2.6.1).
Data analysis	Discovery proteomic data were analysed for pathway enrichment using IPA (QIAGEN Inc., https://www.qiagenbioinformatics.com/products/ingenuitypathwayanalysis). Gene Ontology (GO) annotations were extracted using PANTHER bioinformatics tool (www.pantherdb.org). Most of the statistical analyses were performed in Graphpad Prism (v.9) unless otherwise stated in the methods section.

For manuscripts utilizing custom algorithms or software that are central to the research but not yet described in published literature, software must be made available to editors and reviewers. We strongly encourage code deposition in a community repository (e.g. GitHub). See the Nature Portfolio guidelines for submitting code & software for further information.

Data

Policy information about availability of data

All manuscripts must include a data availability statement. This statement should provide the following information, where applicable:

- Accession codes, unique identifiers, or web links for publicly available datasets
- A description of any restrictions on data availability
- For clinical datasets or third party data, please ensure that the statement adheres to our policy

The raw mass spectrometry proteomics data have been deposited to the ProteomeXchange Consortium via the PRIDE partner repository with the dataset identifier PXD061666 and 10.6019/PXD061666. Source data are provided as a Source data file.

Research involving human participants, their data, or biological material

Policy information about studies with human participants or human data. See also policy information about sex, gender (identity/presentation), and sexual orientation and race, ethnicity and racism.

Reporting on sex and gender	n/a
Reporting on race, ethnicity, or other socially relevant groupings	n/a
Population characteristics	n/a
Recruitment	n/a
Ethics oversight	n/a

Note that full information on the approval of the study protocol must also be provided in the manuscript.

Field-specific reporting

Please select the one below that is the best fit for your research. If you are not sure, read the appropriate sections before making your selection.

☒ Life sciences ☐ Behavioural & social sciences ☐ Ecological, evolutionary & environmental sciences

For a reference copy of the document with all sections, see nature.com/documents/nr-reporting-summary-flat.pdf

Life sciences study design

All studies must disclose on these points even when the disclosure is negative.

Sample size	For discovery proteomics, ubiquitinated proteins and lipid binding proteins 2 biological replicates per condition were used. For functional assays such as ATP production and glycolytic rates 3 biological replicates per condition were used due to the particulars of the instrument specification, and assays repeated at least twice.
Data exclusions	Discovery proteomics identifications and intensities were exported and variables with a confidence score of less than 15 and only one unique peptide were excluded.
Replication	Functional assays were extensively optimised and repeated
Randomization	Allocation of samples was not random. Cells were treated with DMSO and lyso-lipids. For intstrumental analyses samples were ran in random order.
Blinding	V.N. performed treatments, data collection and analysis making blinding impossible

Reporting for specific materials, systems and methods

We require information from authors about some types of materials, experimental systems and methods used in many studies. Here, indicate whether each material, system or method listed is relevant to your study. If you are not sure if a list item applies to your research, read the appropriate section before selecting a response.

Materials & experimental systems

n/a	Involved in the study
☒	☐ Antibodies
☒	☐ Eukaryotic cell lines
☒	☐ Palaeontology and archaeology
☒	☐ Animals and other organisms
☒	☐ Clinical data
☒	☐ Dual use research of concern
☒	☐ Plants

Methods

n/a	Involved in the study
☒	☐ ChIP-seq
☒	☐ Flow cytometry
☒	☐ MRI-based neuroimaging

Plants

Seed stocks	n/a
Novel plant genotypes	n/a
Authentication	n/a
